# Supplementary material for: Low-Frequency Raman Spectroscopy of Few-Layer 2H-SnS2
Source: Sci Rep. 2018 Jul 5;8:10194. doi: 10.1038/s41598-018-28569-6 (PMC6033902; doi:10.1038/s41598-018-28569-6)
Supplement: Supplementary file 1 — Supplementary Information [file 41598_2018_28569_MOESM1_ESM.pdf]

Supplementary Information for

Low-frequency Raman spectroscopy of few-layer 2H-SnS_2

Tharith Sriv[1,2], Kangwon Kim[1] and Hyeonsik Cheong[1,*]

[1]Department of Physics, Sogang University, Seoul 04107, Korea
[2]Department of Physics, Royal University of Phnom Penh, Cambodia
*Corresponding author: hcheong@sogang.ac.kr

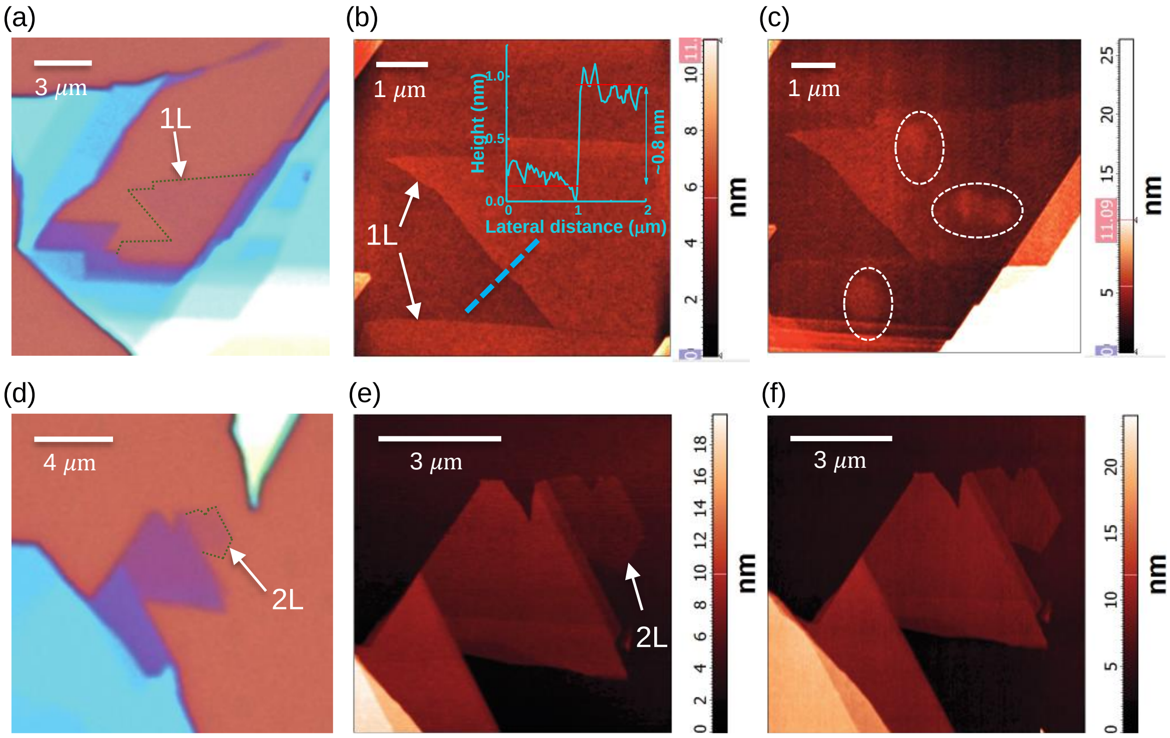

Figure S1. (a, d) Optical images of mechanically-exfoliated 2H-SnS_2 samples on a SiO_2/Si substrate. Dotted outlines indicate the boundaries of monolayer (1L) area. (b, e) Corresponding atomic force microscope (AFM) images of the samples measured before Raman measurements. Inset shows the AFM height profile of the 1L 2H-SnS_2 sample. (c) AFM image of the sample in (a) measured a few hours after the Raman measurements in air using 100 μW of laser power. Damaged areas are indicated. (f) AFM image of the sample in (d) that was measured a week after Raman measurements in vacuum using 100 μW of laser power. No apparent damages are observed.
